# Detection of Dinophysistoxin-1 in Clonal Culture of Marine Dinoflagellate *Prorocentrum foraminosum* (Faust M.A., 1993) from the Sea of Japan

**DOI:** 10.3390/toxins7103947

**Published:** 2015-09-28

**Authors:** Polina A. Kameneva, Kseniya V. Efimova, Viacheslav G. Rybin, Tatiana Y. Orlova

**Affiliations:** 1 A.V. Zhirmunsky Institute of Marine Biology of the Far Eastern Branch of the Russian Academy of Sciences, Palchevskogo St., 17, Vladivostok 690041, Russia; E-Mails: xengen88@gmail.com (K.V.E.); vgrybin@yahoo.com (V.G.R.); torlova06@mail.ru (T.Y.O.); 2 Far Eastern Federal University, Suhanova St., 8, Vladivostok 690950, Russia

**Keywords:** *Prorocentrum foraminosum*, DTX-1, molecular identification, HPLC-HRMS, Russia

## Abstract

For the first time the presence of dinophysistoxin-1 (DTX-1) in a culture of *Prorocentrum foraminosum* was revealed in cells and in the culture medium. The clone was isolated from coastal waters of the Sea of Japan and identified by molecular analyses of SSU and D1/D2 regions of LSU rDNA. The concentration of DTX-1 in cells was 8.4 ± 2.5 pg/cell and, in cell-free media, 27.9 ± 14.7 µg/L. The toxin presence was confirmed by HPLC with high-resolution tandem mass-spectrometry.

## 1. Introduction

The problem of phycotoxins is important for the Russian coastal waters due to the development of mariculture and addressing human health issues [1,2,3]. In order to reveal sources and origin of phycotoxins in marine invertebrates of the far-eastern seas of Russia, the Institute of Marine Biology (FEB RAS) has created the Center of Harmful Algal Bloom (HAB) monitoring. Based on the monitoring data, the most negative effects on shellfish have been recorded from diarrheic shellfish poisoning (DSP) [3,4].

Some species of the genus *Prorocentrum* are known to produce okadaic acid (OA), dinophysistoxins (DTX)-1,-2,-4, other OA derivates, prorocentrolides [5,6], borbotoxins [7], and ichtiotoxin [8,9,10]. In the USA [11] and Canada [12,13], these species, in particular *P. lima*, are considered as one of the main organisms responsible for DSP outbreaks. Moreover, some species, such as *P. rhathymum*, have been recently shown to be toxic [14,15]. Therefore, the Russian HAB monitoring program gives much attention to monitoring of *Prorocentrum* species, their abundance, and potential toxin production.

In June 2013, a clone culture of *P. foraminosum*, first described by M.A. Faust from Twin Cays, Belize [16], was isolated from the coastal waters of the Sea of Japan. To the best of our knowledge, *P. foraminosum* has not yet been tested on the presence of DSP toxins; therefore, its ability to produce toxins is unknown. The goal of this research was to analyze the *P. foraminosum* culture for the potential production of three “parent” DSP toxins: okadaic acid (OA), dinophysistoxin-1 (DTX-1), and dinophysistoxin-2 (DTX-2). Sulfated analogs and diol-esters were shown to be short-living in the environment [17] or hydrolyzed by shellfish releasing the “parent” toxins [18]. Therefore, the estimation of “parent” toxins is important for the primary estimation of *P. foraminosum* potential to be responsible for DSP contamination of the shellfish in the Sea of Japan.

## 2. Results and Discussion

**Figure 1:**
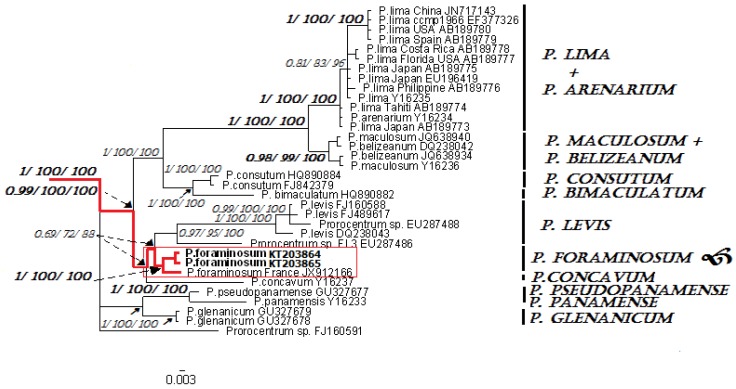
Consensus maximum-likelihood tree based on nuclear SSU rDNA sequences of several members of *Prorocentrum*. Posterior probability, maximum-likelihood, and neighbor-joining bootstrap values <0.5/50/50 are shown from left to right (BI/ML/NJ). Scale bar represents the number of nucleotide substitutions/site.

**Figure 2:**
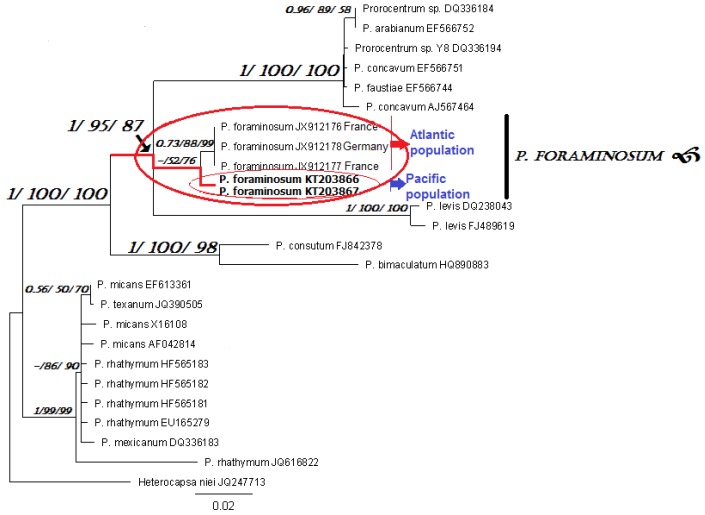
Consensus maximum-likelihood tree based on nuclear D1/D2 region of LSU rDNA sequences of several members of *Prorocentrum*. Posterior probability, maximum-likelihood, and neighbor-joining bootstrap values <0.5/50/50 are shown from left to right (BI/ML/NJ). Scale bar represents number of nucleotide substitutions/site.

Species identification based on the molecular analyses showed that the SSU rDNA gene sequences of the analyzed clones matched with *P. foraminosum* (JX912166 Groix Island, France) with 99% sequence similarity (Figure 1). The D1/D2 region of LSU rDNA sequences were the closest to those of *P. foraminosum* (JX912176–JX912178 Groix Island and Sylt Island) with 95% sequence similarity (Figure 2). For the first time, we obtained the D8/D10 of LSU rDNA and ITS1-5.8S rDNA-ITS2 for *P. foraminosum*. Since only SSU and D1/D2 of LSU for *P. foraminosum* are published in GenBank, the comparative analyses based on the D8/D10 of LSU rDNA and ITS1-5.8S rDNA-ITS2 were not possible.

Thus, we have confirmed that the investigated strain PrRUS_7 of the genus *Prorocentrum* belongs to *P. foraminosum* species. Phylogenetic analyses based on posterior probability, maximum-likelihood and neighbor-joining bootstrap methods have shown clustering of the Pacific *P. foraminosum* (KT203864–KT203865 and KT203866–KT203867, respectively) with the Atlantic *P. foraminosum* sequences in the same clade and clearly separated from other members of the genus *Prorocentrum* (with branch supports: 1/100/100 on SSU and 1/95/87 on D1/D2 of LSU). Intraspecific sequence divergence between Pacific and Atlantic species at the 1% level on SSU and 5% level on LSU might show an inter-population (geographic) variation.

Fluorescent derivatives ((7-methoxy-2-oxo-2*H*-chromen-4-yl)methyl (M-7-MC) derivatives) of “parent” toxins and internal standard 3,12-dihydroxycholan-24-oic acid (deoxycholic acid, DCA), obtained by the reaction of 4-(bromomethyl)-7-methoxy-2*H*-chromen-2-one (4-BrM-7-MC) with the free carboxyl-containing compounds in extracts, were tested by HPLC-FLD and HPLC-HRMS. HPLC-FLD analysis of the standard solutions’ mixture revealed the retention times of M-7-MC derivatives of “parent” toxins under the current chromatographic conditions: OA (7.95 min), DTX-2 (8.57 min), DTX-1 (10.70 min), and the internal standard DCA (11.22 min). These data are in agreement with the previous research by Marr *et al.* [19], where the same fluorescent label was used. The relative elution order of “parent” toxins remained the same.

**Figure 3:**
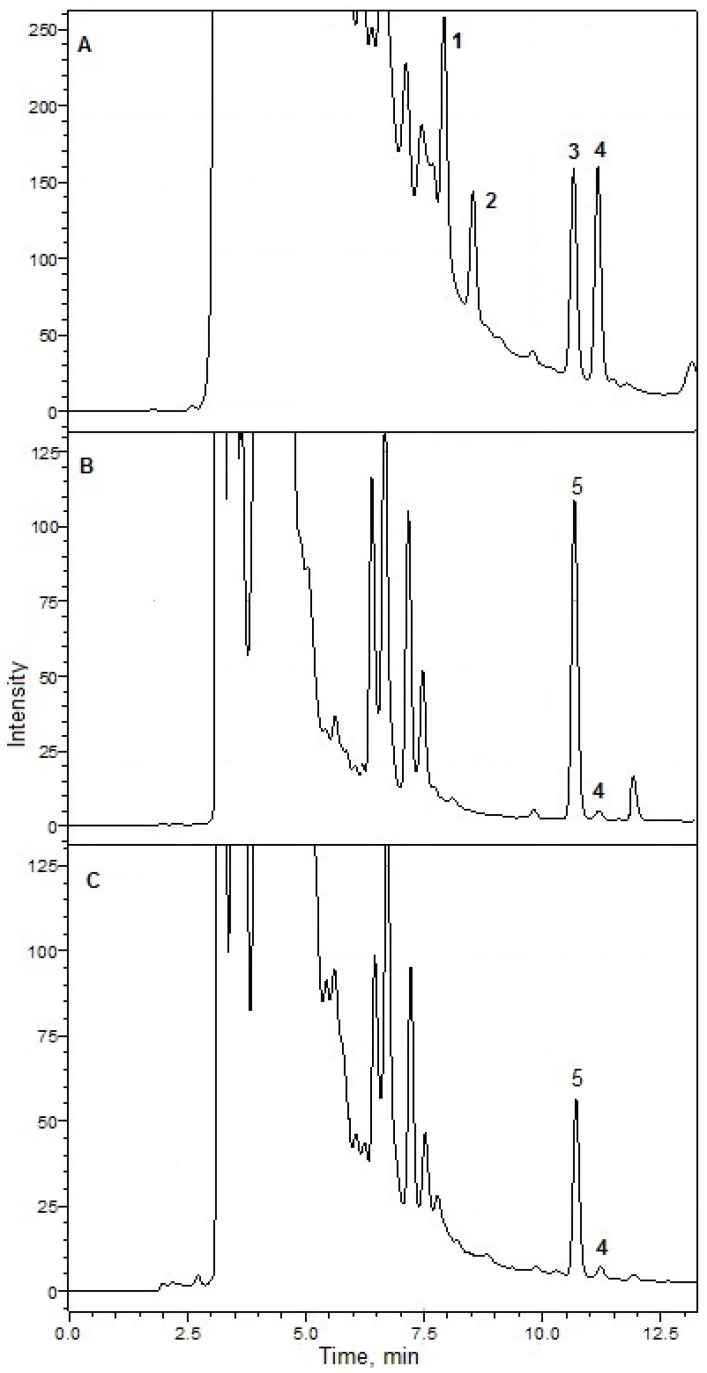
Chromatograms obtained by the HPLC-FLD analyses of the derivatized by 4-BrM-7-MC samples. (**A**) standard solution of OA (17 µM; 50 µL), DTX-1 (18.5 µM; 50 µL), DTX-2 (9.5 µM; 50 µL), DCA (20.3 µM; 50 µL); (**B**) extract of *P. foraminosum* cells with DCA (5.075 µM; 25 µL); (**C**) extract of *P. foraminosum* cells free media with DCA (5.075 µM; 25 µL). Numbers: **1**—OA, **2**—DTX-2, **3**—DTX-1, **4**—DCA, **5**—unknown compound.

In an analysis of cells and cell-free media extracts, the presence of DTX-1 and DCA was shown by matching the retention times (Figure 3A). In the extracts of *P. foraminosum* cells (Figure 3B) and cell-free media (Figure 3C), the unknown compound (5) with the retention time of DTX-1 was observed.

4-BrM-7-MC is rarely used for fluorescent labeling in the analysis of okadaic acid and related toxins in algal cells [19], and the particular derivatization method was not previously tested on the extracts of algal cells and media. Using the HPLC technique with a high-resolution tandem mass-spectrometry (HPLC-HRMS), we have identified the unknown compound that formed peak 5 (Figure 3).

**Figure 4:**
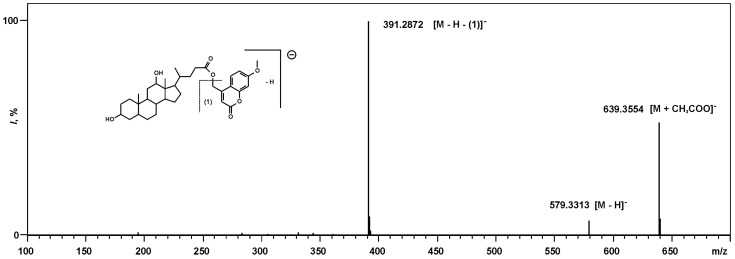
APCI-MS of M-7-MC derivative of DCA.

In order to reveal the ionization pattern of M-7-MC derivatives, the initial analysis of (7-methoxy-2-oxo-2*H*-chromen-4-yl) methyl-3,12-dihydroxycholan-24-oate (M-7-MC derivative of DCA) was performed. Results of atmospheric pressure chemical ionization (APCI) MS analysis of this compound showed the presence of three types of negative ions in the spectrum (Figure 4) with *m/z* 639.3554, 579.3313 and 391.2872. These *m/z* values correspond to following composition of the ions: [C_37_H_51_O_9_]^−^ (calculated 639.3539), [C_35_H_47_O_7_]^−^ (calculated 579.3327) and [C_24_H_39_O_4_]^−^ (calculated 391.2854), respectively. Ions with the composition [C_37_H_51_O_9_]^−^ formed as a result of the acetate anions’ addition to the M-7-MC derivative of DCA and corresponded to acetylated cluster ions with the composition [M + CH_3_COO]^−^. Ions with the composition [C_35_H_47_O_7_]^−^ corresponded to deprotonated quasi-molecular ions [M − H]^−^ of the M-7-MC derivative of DCA. Ions with the composition [C_24_H_39_O_4_]^−^ corresponded to anions of DCA, formed after the loss of the M-7-MC group from quasi-molecular ions.

**Figure 5:**
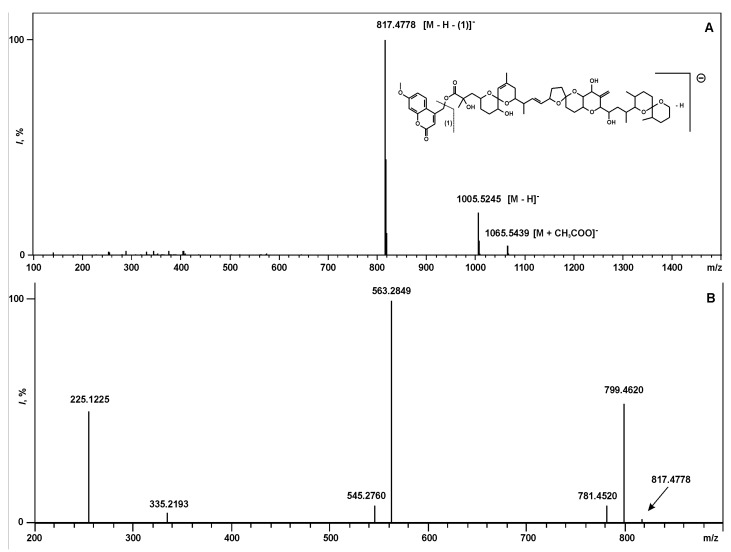
(**A)** APCI-MS of unknown component (Figure 3B,C, peak 5); (**B)** MS^2^ of parent ions with *m*/*z* 817.4778.

The component of the reaction mixture with a retention time 10.70 min (Figure 3B,C, peak **5**) showed an APCI-MS pattern similar to the APCI-MS pattern of the M-7-MC derivative of DCA. The quasi-molecular ions, and ions formed by the addition of acetate anions to the molecules of the component, as well as the ions formed by elimination of M-7-MC group of quasi-molecular ions, were present in the spectrum (Figure 5A). Ions with *m/z* 1065.5439 differed from ions with *m/z* 1005.5245 on 60.0194 a.m.u., which corresponds to monoisotopic molecular mass of acetic acid (calculated 60.0211), and it is possible that the ions formed by the addition of acetic acid anions to molecules of the component [M + CH_3_COO]^−^. Ions with *m/z* 1005.5245 corresponded to negative deprotonated quasi-molecular ions [M − H]^−^ formed from molecules with a composition C_56_H_78_O_16_ (calculated 1005.5217 for the deprotonated molecules). This corresponds to a molecular formula with the elemental composition of M-7-MC derivative of DTX-1. More intensive ions with *m/z* 817.4778 formed by elimination of neutral molecules (M-7-MC groups without a hydrogen atom) with a value of their monoisotopic molecular mass 188.0467 a.m.u. (calculated 188.0473). We assume that these ions are deprotonated DTX-1 anions. MS^2^ spectrum of precursor ions with *m/z* 817.4778 (Figure 5B) showed a similar pattern to the previously described MS^2^ spectral data for the deprotonated DTX-1 anions [20]. Thus, the unknown substance of the reaction mixture was determined as M-7-MC derivative of DTX-1. By finding a similar ionization pattern of internal standard and DTX-1 in the sample and by comparison of the known ionization patterns for DTX-1, we can directly confirm the presence of DTX-1 in the extracts of cells and cell-free media of *P. foraminosum*.

The average concentration of cells in the cultures, calculated using a 1 mL Sedgewick-Rafter counting chamber by light microscopy, was 4191 ± 657 cells/mL. For the analysis, 40 mL of culture was used. The concentration of DTX-1 per cell accounted for an average of 8.4 ± 2.5 pg/cell (Figure 3B). However, the thecae without any content inside were found while counting the cell number and the concentration of those thecae was significant: 3427 ± 1145 cells/mL. We suppose that the toxins that might have been contained in those cells were released in the media and we considered it important to estimate the concentration of toxins in the cell-free media. The results of analysis showed the presence of DTX-1 in a concentration of 27.9 ± 14.7 µg/L (Figure 3C). No extracts of cells or cell-free media contained substances that matched with the retention times of M-7-MC derivatives of OA and DTX-2.

Currently, 10 species of genus *Prorocentrum* are known to produce various toxins [6]. However, not all species produce okadaic acid and its analogues. For example, *P. borbonicum* is known as a borbotoxins producer [6], and *P. concavum* is capable of producing unidentified ichthyotoxin [8,9,10]. The potential production of DSP toxins by other species of genus *Prorocentrum* still require more precise testing, as *P. faustidae* and *P. maculosum* might have been misidentified [6]. The data for toxicity of *P. levis* also remains questionable due to the only evidence being from personal communication [6], and the research of [21,22] did not prove it. Therefore, the data about these species are not taken into account. The data for other species are summarized in Table 1.

**Table 1:** Okadaic acid toxins production by various *Prorocentrum* species.

Species	Toxin	“Location”	Concentration	Reference
*P. lima*	OA	field cells	4–6.4 pg/cell	[23]
		algal sample	21 ng/mg	[24]
		culture cells	1.3–26 pg/cell	[8,25,26]
		culture media	25 µg/L	[27]
	DTX-1	culture cells	4–14.3 pg/cell	[25,26]
		culture media	25 µg/L	[27]
*P. belizeanum*	OA	culture cells	12.45 pg/cell	[28]
*P. hoffmannianum*	OA	culture cells and media	500–800 µg/L	[29]
*P. concavum*	OA	culture cells	10–15 pg/cell	[8]
*P. rhathymum*	OA	culture media	0.153 µg/L	[14,15]
*P. foraminosum*	DTX-1	culture cells	8.4 ± 2.5 pg/cell	present study
		culture media	27.9 ± 14.7 µg/L	

According to the information about toxin production by *Prorocentrum* species, only *P. lima* is known to produce DTX-1 (Table 1). The toxin production by *P. foraminosum* cells in culture is within the range of toxin production by *P. lima*. In our samples it was higher than in samples collected in Spain [25], but lower than in some samples from Australia [26]. As to the concentration of toxins in media, the value for *P. foraminosum* was slightly higher than that for *P. lima* [27]. Thus, for the first time, we have demonstrated that the clone culture of *P. foraminosum* isolated from the coastal waters of the Sea of Japan was capable of producing moderate concentrations of DTX-1.

Information about toxin production by *Prorocentrum* species is quite reliable, but still hardly comparable due to variability of samples tested in field and in culture. As for culture, we have obtained data for cells and for media. Due to estimation of environmental conditions in culture, we cannot recommend extending the data for culture samples to the field samples. Thus, field samples and concentrations of sulfated analogs and diol-esters in culture and field samples need further testing.

## 3. Experimental Section

### 3.1. Reagents and Standards

All solvents were of HPLC grade. Standard solutions of okadaic acid, dinophysistoxin-1, and dinophysistoxin-2 were purchased from the certified reference material program (CRMP) of the Institute for Marine Biosciences, National Research Council (Canada). Components for K medium were from K medium Kit, 50 L Provasoli-Guillard National Center for Marine Algae and Microbiota NCMA. 4-(bromomethyl)-7-methoxycoumarine (Al 235202), 18-crown-6 ether (Fl2815), anhydrous potassium carbonate (P5833), deoxycholic acid (Fl 30960) were from Sigma Aldrich.

### 3.2. Microalgae Collection

Samples of epiphytes were collected from the macrophyte *Neorhodomela aculeate* using scuba at 1 m depths in the Zhitkova Bay of Bosphor Vostochny Gulf, the Sea of Japan (43.021403 N, 131.930293 E) on 10 of June 2013. Collection and concentration of epiphyton samples were made according to the method described earlier [30]. The macrophyte was placed in screw cap plastic jars under the water. Then jars with macrophytes were vigorously shaken for 1 min to dislodge the dinoflagellates from the macrophyte surface. The remaining epiphytes were dislodged from the macrophytes by thoroughly rinsing with filtered seawater. The cell suspensions were sieved through a 120-μm screen and then concentrated on a 20-μm sieve.

### 3.3. Isolation, Culturing and Cell Number Estimation

Single-cell isolation was made by sterile micropipette and individual cells were inoculated into separate wells of an eight-chamber culture tray containing K enriched seawater medium made from autoclaved seawater with 34% salinity. Incubation of cultures was in the incubation chamber KBW 400 (Binder, Germany) at 20 °C on a 12:12 h light:dark cycle and illumination of 39 µmol/m^−2^ s^−1^. After several divisions, individual cells were transferred sequentially to petri dishes and 50-mL glass flasks with the same media and incubation conditions to obtain monoclonal non-axenic culture. Twenty clones of *Prorocentrum* cf. *foraminosum* were obtained and some of them are kept in the Center of marine microbiota culturing of IMB FEB RAS (www.imb.dvo.ru/misc/toxicalgae/index.htm). The strain PrRUS_7 was grown successfully and provided necessary volume for the analysis.

For cell number estimation the culture was well-mixed in a culture flask. 1 mL of culture was removed directly from the flasks, 4 mL of cell-free K medium was added to the sample for dilution, then it was fixed with a 0.2% v/v acidic Lugol’s solution, and enumerated for *Prorocentrum* cell concentrations using a 1 mL Sedgewick-Rafter chamber and light microscopy at 100× magnification in triplicate. The dilution factor of five was included in calculations.

### 3.4. Molecular Identification of Species

The clonal culture was sequenced in the ribosomal gene pool of nuclear DNA. Total DNA was extracted as described by Montero-Pau *et al*. [31]. The DNA amplification and DNA sequencing of PCR products of the small subunit (SSU rDNA), D1/D2 region of the large subunit (LSU rDNA), and ITS1-5.8S rDNA-ITS2, as well as molecular-phylogenetic analysis were carried out as described previously [32], except that the cloning of LSU fragments prior to sequencing was omitted from the experiment. The DNA amplification and sequencing of PCR products of the D8/D10 of LSU rDNA were carried out using the oligonucleotide primers FD8 (5′-GGATTGGCTCTGAGGGTTGGG-3′) and RB (5′-GATAGGAAGAGCCGACATCGA-3′) [33] with PCR conditions as for ITS1-5.8S rDNA-ITS2 [32]. The sequences were deposited in GenBank, with accession numbers KT203864–KT203871, and were compared with available GenBank sequences using the BLAST software program (NCBI).

### 3.5. Toxin Extraction

#### 3.5.1. Cells

Forty mL of well-mixed culture on stationary phase were transferred to a 50 mL screw-cup tube and centrifuged at 4000 RCF for 30 min. Supernatant containing almost no cells was transferred to a clean tube and 1.5 mL of 80% methanol was added to the pellet. The pellet was sonicated for 5 min in an ice bath and vortexed for 1 min followed by centrifugation at 4000 RCF for 10 min. The supernatant was transferred to a glass tube with a screw cup. The residue was extracted again by 1 mL of 80% methanol and extracts were combined. The analysis was repeated in five replicates.

#### 3.5.2. Media

Forty mL of media were filtered through the syringe-driven filter 0.22 nm (Merck Milipore Ltd., Ireland) for cell removal. Media samples were loaded on solid phase extraction (SPE) cartridge (Supelco, USA) containing 0.3 mg of C-18 sorbent previously conditioned with methanol and distilled water. DSP toxins were eluted by 2 mL of methanol. The extract was diluted with 0.5 mL of water transferred to glass tube with a screw cup. The analysis was repeated in five replicates.

#### 3.5.3. General

Two mL and five hundred µL of 80% methanol extract of cells and media were further extracted twice with 2.5 mL of hexane by vigorous shaking for 1 min. Hexane layers were discarded. Five hundred µL of water and 75 µL of concentrated HCl were added to the aqueous methanol and extracted twice with 3 mL of chloroform. The pooled chloroform extracts were evaporated to dryness in vacuum at 35 °C. The residue was dissolved in 100 µL of mixture acetonitrile/chroloform (1:9). The whole extract was transferred to reaction vial for derivatization.

### 3.6. Derivatization and Purification

Derivatization and purification were carried out by a slightly modified method of Imbs *et al*. [34]. The work solutions of 15 mg/mL of 4-(bromomethyl)-7-methoxycoumarine (4-BrM-7-MC) and 100 mg/mL of 18-crown-6 ether (CrEth) in the mixture of acetonitrile/chloroform (1:9) were prepared immediately before reaction. In a reaction vial 100 μL of extract, 200 μL of 4-Br-7-MMC and 50 µL of CrEth were mixed. Approximately 15 mg of anhydrous potassium carbonate powder was added. Reaction mixture was incubated at 78 °C for two hours. To stop the reaction, the reaction mixture was cooled, than it was filtered and evaporated to dryness under the steam of argon. The residue was dissolved in 500 µL of ethyl acetate/benzene mixture (15:85) and transferred to an SPE cartridge (Diapack C, BioChimMack, Russia) containing 1 mg of silica gel, previously conditioned with 3 mL of methanol and 3 mL of ethyl acetate/benzene mixture (15:85). After wash step with 3 mL ethyl acetate/benzene mixture (15:85), M-7-MC ethers were eluted by 3 mL of ethyl acetate. The final fraction was evaporated to dryness in vacuum at 35 °C and re-dissolved in 350 μL of acetonitrile for HPLC analysis.

### 3.7. High Performance Liquid-Chromatography with Fluorescent Detection (HPLC-FLD)

HPLC-FLD analysis was performed on a LC-20A Prominence chromatograph with a fluorescent detector RF-10A_XL_ (Shimadzu, Japan). The separation was performed through the YMC^®^ C18 column (250 mm; 4.6 mm i.d.; 5 µm part size, YMC Co., Ltd., Japan) with a guard column YMC ProC18 (10 mm; 4 mm i.d.; 5 µm part size, YMC Co., Ltd., Japan) at 40 °C. Signal recording was carried out at an excitation wavelength of 325 nm and an emission wavelength of 391 nm. The injection volume was 2 µL. The mobile phase consisted of acetonitrile (A) and water (B). The following elution program was used, with a flow rate of 1 mL/min: eluent A–eluent B (80:20, v/v) up to 2 min, then eluent А up to 17 min and then eluent A–eluent B (80:20, v/v) up to 80 min.

### 3.8. Toxin Concentration Estimation

Deoxycholic acid (DCA) was used as an internal standard [35]. DCA solution in methanol (25 µL, 5.075 µM) was added to each sample in the beginning of the extraction process, which was corresponding to 50 ng DCA per sample. Estimation of the toxin concentration was based on the proportion:

C_(DCA)_/S_(DCA)_ = C_(toxin)_/S_(toxin)_ (1)

where C is the concentration of DCA or toxin in the sample and S is the area of the peak on the chromatogram. The total toxin concentration of the cell pellet was divided by the number of cells to get the toxin concentration per cell. The total toxin concentration in the medium was divided by the volume of medium tested to get concentration per mL.

### 3.9. High Performance Liquid Chromatography-High Resolution Mass-Spectrometry (HPLC-HRMS)

The HPLC-HRMS analysis was performed on a LCMS-IT-TOF (Shimadzu, Japan) system equipped with a LC-20A Prominence chromatograph and an ion-trap/time-of-flight mass spectrometer. Separation occurred through the Ascentis^®^ C18 (100 mm; 2.1 mm i.d.; 3 µm part size) at 40 °C. The sample volume was 20 µL. The mobile phase consisted of acetonitrile with 0.1% of acetic acid (A) and water with 0.1% of acetic acid (B). The following elution program was used, with a flow rate of 0.25 mL/min: eluent A–eluent B (80:20, v/v) up to 2 min, then eluent А up to 17 min and then eluent A–eluent B (80:20, v/v) up to 80 min. The range of detection was *m/z* 100–1500 (APCI, negative-ion detection). The potential in the ion source was −3.5 kV. The drying gas (N_2_) pressure was 25 kPa. The nebulizer gas (N_2_) flow rate was 2 L/min. The interface temperature was 300 °C. For MS^2^ experiments the range of detection was *m/z* 50–850. The collision gas (Ar) pressure was 0.003 Pa (50% from total pressure in the collision cell).

## 4. Conclusions

In this research, an isolated clonal culture of *Prorocentrum* from the coastal waters of the Sea of Japan was identified as *P. foraminosum* by molecular-genetic analyses of SSU rDNA and D1/D2 region of LSU rDNA and phylogenetic analyses based on the gene sequences of members of the genus *Prorocentrum*. For the first time, the research showed that *P. foraminosum* is capable of producing moderate concentrations of DTX-1 under culture conditions. The presence of the toxin in cells and cell-free media extracts was confirmed by HPLC-HRMS. Still, the ability of this species to produce toxins in the field needs testing. The precise toxin profile of sulfated analogs and diol esters should also be considered.
